# Application of Machine Learning Models in Predicting Vibration Frequencies of Thin Variable Thickness Plates

**DOI:** 10.3390/ma19010205

**Published:** 2026-01-05

**Authors:** Łukasz Domagalski, Izabela Kowalczyk

**Affiliations:** Department of Structural Mechanics, Lodz University of Technology, Politechniki 6, 93-590 Lodz, Poland

**Keywords:** surrogate model, machine learning, variable thickness plates, vibrations, natural frequencies

## Abstract

This study investigates the application of machine learning (ML) techniques for predicting vibration frequencies of thin rectangular plates with variable thickness. Traditional optimization methods, such as genetic algorithms, require repeated solutions of the plate vibration eigenproblem using finite element (FE) analysis, which is computationally expensive. To reduce this cost, a surrogate model based on artificial neural networks (ANNs) is proposed as an efficient alternative. The dataset includes variations in plate geometry, boundary conditions, and thickness distribution, encoded numerically for model training. ANN architecture and hyperparameters—such as the number of hidden layers, neurons per layer, and activation functions—were systematically tuned to achieve high prediction accuracy while avoiding overfitting. Data preprocessing steps, including standardization and scaling, were applied to improve model stability. Performance was evaluated using metrics such as RMSE and R^2^. The results demonstrate that ANNs can accurately predict eigenvalues with significantly reduced computational effort compared to FE analysis. This approach offers a practical solution for integrating machine learning into structural optimization workflows.

## 1. Introduction

This study focuses on developing surrogate machine learning models for predicting vibration frequencies of thin rectangular plates with variable thickness distribution. The approach uses machine learning (ML) techniques to replace computationally expensive finite element analyses (FEA) in optimization processes, offering a significant advancement in structural dynamics prediction and design optimization workflows.

Machine learning (ML) is increasingly used in structural vibration analysis. Various network architectures aim to improve prediction accuracy while reducing computational cost [1,2,3]. Multiple key studies have shown that neural networks can effectively predict vibration frequencies in plate structures [4,5,6]. Reddy [7] demonstrated the effectiveness of artificial neural networks (ANNs) in predicting natural frequencies of laminated composite plates, achieving high accuracy with minimal relative error compared to finite element analysis results. Seba and Kebdani [8] combined finite element analysis with neural networks to predict frequencies of laminated plates with cutouts, achieving high accuracy on 520 configurations. Altabey [8] focused on the application of ANNs in predicting the natural frequency of basalt fiber reinforced polymer laminated, variable thickness plates. The author has found that the finite strip transition matrix approach is very effective in studying the changes in plate natural frequencies due to intermediate elastic support, but the method’s difficulty in terms of many calculations with a large number of iterations is the main drawback of the method. In [9], the authors present a comparative analysis between two methods: the state-space-based differential quadrature method and ANN for different boundary conditions of functionally graded (FG) annular plates. ANN was employed to model the FG annular plates and to predict the effects of different parameters on the natural frequency of the plates.

There are also numerous works where researchers tested ML techniques on different structural elements. Several researchers have extended machine learning applications to various beam structures with promising results [10,11,12,13]. In [14], the authors present a novel and effective ANN-differential evolution approach to solve the problem of the material distribution optimization of bidirectional functionally graded (BFG) beams under free vibration. In this methodology, ANN is utilized as an analyzer to predict responses of BFG beams instead of directly solving eigenvalue problems via time-consuming FEAs. Yildirim [15] deals with the estimation of free vibration characteristics of beams made of functionally graded material. The proposed ANN model can predict the natural frequencies without the need for a solution of any differential equation or time-consuming experimental processes. Das [16] conducted a comprehensive study on predicting natural frequencies of beams using regression machine learning models and obtained results indicating that the considered regression ML models are effective in predicting the natural frequencies of beam structures. Avcar and Saplıoğlu [17] developed ANN models for estimating natural frequencies of prismatic steel beams with various geometrical characteristics under four different boundary conditions, successfully creating 36 different models and achieving excellent agreement between ANN predictions and theoretical results with minimal computational requirements. Furthermore, Liu and Yang [18] introduced an innovative approach using convolutional neural networks (CNN) for vibration frequency prediction directly from image sequences, demonstrating that frequency prediction can be achieved using a single feed-forward CNN without explicit vibration signal extraction, offering a non-contact measurement alternative for beam structures.

Advances in deep learning have improved vibration prediction methods. For example, Van Delden et al. [19] proposed the Frequency–Query Operator network architecture specifically designed for predicting vibration patterns of plate geometries given specific excitation frequencies. Their method outperforms DeepONets, Fourier Neural Operators, and more traditional neural network architectures and can be used for design optimization. Timchenko and Osetrov [20] used CNNs specifically tailored for predicting natural frequencies of composite plates with non-canonical shapes, demonstrating that CNN architecture can effectively capture complex geometric features that influence vibrational behavior. In [21], the authors developed a sophisticated neural network-based prediction model to investigate the combined influence of temperature and moisture on vibration characteristics of skew laminated composite sandwich plates. This work highlighted the importance of considering environmental factors in vibration prediction models, particularly for composite structures operating in harsh conditions.

In parallel to vibration-focused studies, interpretable machine-learning surrogates have been successfully applied in materials and mechanics contexts, for example, through gradient-boosted trees with SHAP-based explanations for metallic glasses [22] and active-learning frameworks for FeNiCoAlTa alloy design [23]. These works highlight that non-black-box models and adaptive sampling strategies can provide both high predictive accuracy and physical insight, and they motivate the broader use of ML surrogates in structural and materials optimization. In this study, however, we restrict our attention to ANN-based regression models and focus on their accuracy and computational efficiency for plate-vibration prediction.

The main focus of this study is to identify the most suitable regression model based on artificial neural networks. Multiple parameters can influence model performance and require careful optimization. First, data preprocessing transformers that perform standardization and/or scaling of input data must be properly configured. Subsequently, depending on the chosen regressor, the architecture of ANNs or decision trees becomes equally critical as the preprocessing steps mentioned above. Furthermore, to prevent model overfitting, the optimal size of the training dataset must be determined through systematic investigation. This work primarily focuses on the selection of the best-fitting model and the fine-tuning of its parameters to achieve sufficiently accurate predictions while maintaining reasonable computational efficiency.

## 2. Methods

### 2.1. Theoretical Background

**Figure 1:**
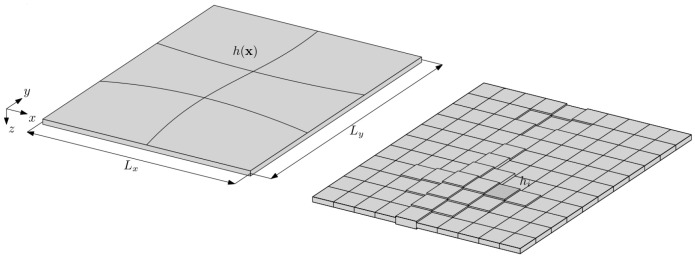
Variable thickness plate and its FE approximation.

Let *Oxyz* be an orthogonal Cartesian coordinate system in the physical space. Considered a structural element in this system is a plate of dimensions *L_x_* × *L_y_*, which are parallel, respectively, to the *Ox* and *Oy* axes. The *Oz* is perpendicular to the mid-surface Π of the plate. The plate is of variable thickness *h* = *h*(*x*, *y*), and it is made of an isotropic linear-elastic material (Figure 1). To simplify the notation, it was assumed that: **x** = (*x*, *y*). Material properties are described by the following constants: Young’s modulus *E*, Poisson’s ratio *ν*, and density *ρ*. The plate is represented by a well-known model according to the Kirchhoff–Love theory for thin plates, with three fundamental kinematic constraints: straight lines normal to the mid-surface remain straight and normal after deformation; the plate thickness remains constant during deformation; and transverse shear deformations are negligible, *ε_zz_* = 0. The variational formulation of fundamental equations for this problem is briefly presented below, according to [24,25,26,27].

The analyzed element (plate) is considered in a certain finite time interval *T*, which is bounded by *t*_0_ and *t*_1_, representing the initial and current moments, respectively, so the action functional is given by the following:

(1) $$A=\int_{t_{0}}^{t_{1}}Ldt,$$

The Lagrangian in Equation (1) is of the following form:

(2) L=K−W−qw+pw,

where w=w(x,t) stands for transverse deflection. In the general case, q=q(x,t) is a transverse load on the surface of the plate. The dissipative force *p* is assumed as follows, where c=c(x) stands for the damping coefficient:

(3) $$p=p(x,t)=c(x)\dot{w}(x,t).$$

The equations of motion can be obtained from the extended principle of stationary action (see [28]), formulated as follows:

(4) $$\delta A=\delta \left(\int_{t_{0}}^{t_{1}}Ldt\right)=\int_{t_{0}}^{t_{1}}\delta Ldt=0.$$

The strain or potential energy of the deformed plate is equal to:

(5) $$W=\frac{1}{2}\underset{\Omega }{∭}\left(\sigma_{xx}\varepsilon_{xx}+\sigma_{yy}\varepsilon_{yy}+2\sigma_{xy}\varepsilon_{xy}\right)d\Omega ,$$

where $\Omega \equiv \left\{(x,z):x\in \prod ,-h(x)/2\leq z\leq h(x)/2\right\}$ denotes a bounded region representing the undeformed plate. Strains *ε* are described by kinematic relations (6) and stresses *σ* by the stress–strain relations (7), according to Kirchhoff–Love theory, they are defined as follows:

(6) $$\begin{array}{l} \varepsilon_{xx}=-z\frac{\partial^{2}w}{\partial x^{2}},\;\;\;\;\varepsilon_{yy}=-z\frac{\partial^{2}w}{\partial y^{2}},\;\;\;\;\varepsilon_{zz}=-z\frac{\partial^{2}w}{\partial z^{2}}=0,\;\;\;\;\varepsilon_{xy}=-z\frac{\partial^{2}w}{\partial x\partial y},\\ \varepsilon_{xz}=\frac{1}{2}\left[\frac{\partial }{\partial z}\left(u-\frac{\partial w}{\partial x}z\right)+\frac{\partial w}{\partial x}\right]=0,\;\;\;\;\varepsilon_{yz}=\frac{1}{2}\left[\frac{\partial }{\partial z}\left(v-\frac{\partial w}{\partial y}z\right)+\frac{\partial w}{\partial y}\right]=0, \end{array}$$

(7) $$\sigma_{xx}=\frac{E}{1-\nu^{2}}\left(\varepsilon_{xx}+\nu \varepsilon_{yy}\right),\;\;\;\;\sigma_{yy}=\frac{E}{1-\nu^{2}}\left(\varepsilon_{yy}+\nu \varepsilon_{xx}\right),\;\;\;\;\sigma_{xy}=\frac{E}{1-\nu^{2}}\varepsilon_{xy},$$

where *ν* stands for Poisson’s ratio. Displacement variables *u* and *v* used in Equation (6) stand for the displacements of a point in the plate along the *x* and *y* axes, respectively. According to Kirchhoff–Love theory, these displacements do not depend on the through-thickness coordinate *z*. In consequence, transverse strain components *ε_xz_* and *ε_yz_* are equal to zero.

Substituting the kinematic relations (6) and stress–strain constitutive laws (7) into the potential energy formulation (5) yields the final strain energy expression (8) as follows:

(8) $$W=\frac{E}{2\left(1-\nu^{2}\right)}\underset{\Omega }{∭}z^{2}\left\{{\left(\frac{\partial^{2}w}{\partial x^{2}}\right)}^{2}+{\left(\frac{\partial^{2}w}{\partial y^{2}}\right)}^{2}+2\nu \frac{\partial^{2}w}{\partial x^{2}}\frac{\partial^{2}w}{\partial y^{2}}+2\left(1-\nu \right){\left(\frac{\partial^{2}w}{\partial xy}\right)}^{2}\right\}d\Omega .$$

Considering only the transverse motion, the kinetic energy of the plate can be expressed as follows:

(9) $$K=\iint_{\prod }\frac{1}{2}\mu (x){\dot{w}}^{2}d\prod ,$$

where the overdot above *w* stands for the derivative taken with respect to time *t* and μ(x) is the mass density of the plate material per unit area as follows:

(10) $$\mu =\mu (x)=\int_{-h(x)/2}^{h(x)/2}\rho dz.$$

Substitution of the aforementioned Equations (1)–(10) and integrating by parts leads to Equation (11):

(11) $$\nabla^{2}(D\nabla^{2}w)-(1-\nu )\left(\frac{\partial^{2}D}{\partial y^{2}}\frac{\partial^{2}w}{\partial x^{2}}-2\nu \frac{\partial^{2}D}{\partial x\partial y}\frac{\partial^{2}w}{\partial x\partial y}+\frac{\partial^{2}D}{\partial x^{2}}\frac{\partial^{2}w}{\partial y^{2}}\right)+c\dot{w}+\mu \ddot{w}=q(x,t),$$

where D=D(x) is varying flexural stiffness, and $\nabla^{2}$ is the Laplace operator, given by Equation (12). The *f*(**x**) stands for a twice-differentiable real-valued function:

(12) $$\nabla^{2}(f(x))=\frac{\partial^{2}(f(x))}{\partial x^{2}}+\frac{\partial^{2}(f(x))}{\partial y^{2}}.$$

Equation (11) constitutes the fourth-order governing equation of motion for the out-of-plane displacement of a thin plate with variable thickness [27].

### 2.2. Finite Element Approach to Linear Plate Vibration Problems

The natural frequencies and mode shapes, representing the plate’s free vibration characteristics, as well as the amplitudes of forced vibrations, can be determined by solving Equation (11) with the finite element method (FEM). The equations were formulated based on [29]. The plate was discretized using four-node rectangular elements.

The generalized equation of motion in FEM dynamics analysis is given by the following:

(13) $$M\ddot{q}(t)+C\dot{q}(t)+Kq(t)=F(t),$$

where **M**, **C**, and **K** are matrices of mass, damping, and stiffness, respectively. The remaining components of Equation (13) depend on time *t*, where **F**(*t*) is the vector of external loads, and **q**(*t*) is the displacement vector. First time derivative of displacement $\dot{q}(t)$ is velocity, and the second derivative $\ddot{q}(t)$ stands for acceleration. The matrices of mass **M**, damping **C**, and stiffness **K** for the entire structure can be defined as follows:

(14) $$M=\sum_{e}M^{\left(e\right)},\;\;\;C=\sum_{e}C^{\left(e\right)},\;\;\;K=\sum_{e}K^{\left(e\right)},$$

where the superscript (*e*) denotes the single finite element *e*. To define all components in a presented formula, let the stress–strain relation be expressed in the following matrix form:

(15) $$\sigma^{\left(e\right)}=D^{\left(e\right)}\varepsilon^{\left(e\right)},$$

where **σ**^(*e*)^ and **ε**^(*e*)^ stand for stress and strain vectors, respectively, **D**^(*e*)^ is the stress–strain relation matrix.

Strain Formulas (6) in a matrix form can be expressed as follows:

(16) $$\varepsilon^{\left(e\right)}={\left[\begin{array}{c} \varepsilon_{xx}\\ \varepsilon_{yy}\\ \varepsilon_{xy} \end{array}\right]}^{\left(e\right)}=-z{\left[\begin{array}{c} \kappa_{xx}\\ \kappa_{yy}\\ \kappa_{xy} \end{array}\right]}^{\left(e\right)}=-z\kappa^{\left(e\right)},$$

where **κ**^(*e*)^ stands for the plate’s curvature. Similarly, stress–strain relations (7) in a matrix form are defined as follows:

(17) $$\sigma^{\left(e\right)}={\left[\begin{array}{c} \sigma_{xx}\\ \sigma_{yy}\\ \sigma_{xy} \end{array}\right]}^{\left(e\right)}=-D^{\left(e\right)}\varepsilon^{\left(e\right)}=-zD^{\left(e\right)}\kappa^{\left(e\right)},$$

where

(18) $$D^{\left(e\right)}=\frac{E}{1-\nu^{2}}\left[\begin{array}{ccc} 1 & \nu & 0\\ \nu & 1 & 0\\ 0 & 0 & (1-\nu )/2 \end{array}\right].$$

Unit shear forces on the edge of the plate element, in the middle plane, replacing the linearly varying stresses at the height of the element, are defined as follows:

(19) $$\begin{array}{l} m^{\left(e\right)}={\left[\begin{array}{c} m_{xx}\\ m_{yy}\\ m_{xy} \end{array}\right]}^{\left(e\right)}=\int_{-h/2}^{h/2}\sigma^{\left(e\right)}zdz=-\int_{-h/2}^{h/2}D^{\left(e\right)}\kappa^{\left(e\right)}z^{2}dz=-{\bar{D}}^{\left(e\right)}\kappa^{\left(e\right)}=-{\bar{D}}^{\left(e\right)}B^{\left(e\right)}v^{\left(e\right)}, \end{array}$$

where

(20) $${\bar{D}}^{\left(e\right)}=\frac{Eh^{3}}{12(1-\nu^{2})}\left[\begin{array}{ccc} 1 & \nu & 0\\ \nu & 1 & 0\\ 0 & 0 & (1-\nu )/2 \end{array}\right].$$

In this case, *h* is also the thickness, but it is constant for individual plate element *e*. **B**^(*e*)^ stands for the linear strain matrix of elasticity of element *e*, and **v**^(*e*)^ is the matrix of geometrical parameters for individual element.

Based on the principle of stationary action (4) presented in Section 2.1, the stress–strain relations (15) and strain–displacement relations (16), the mass, damping, and stiffness matrices in the generalized equation of motion (13) can be expressed as follows:

(21) $$K^{\left(e\right)}=\int_{A^{\left(e\right)}}B^{(e)T}{\bar{D}}^{\left(e\right)}B^{\left(e\right)}dA^{\left(e\right)}.$$

**K**^(*e*)^ is the stiffness matrix for element *e*. **V**^(*e*)^ is a matrix of static parameters for the individual element, and *A*^(*e*)^ stands for the area of the element *e*.

The mass matrix **M**^(*e*)^ and damping matrix **C**^(*e*)^ for individual plate element *e* can be defined as follows:

(22) $$M^{\left(e\right)}=\int_{A^{\left(e\right)}}\rho^{\left(e\right)}N^{T(e)}N^{\left(e\right)}dA^{\left(e\right)},$$

(23) $$C^{\left(e\right)}=\int_{A^{\left(e\right)}}\eta^{\left(e\right)}N^{T(e)}N^{\left(e\right)}dA^{\left(e\right)},$$

where **N**^(*e*)^ is the shape functions vector of element *e*, **ρ**^(*e*)^ is the mass density matrix, and *η*^(*e*)^ is the damping parameter of element *e*. Here, the damping matrix was defined according to the Rayleigh damping model, commonly used in numerous studies [30,31]:

(24) C=αM+βK,

where *α* and *β* are the Rayleigh coefficients.

In the case of forced harmonic vibrations, the external force can be expressed as follows:

(25) $$F(t)=F_{0}(t)\cos(\omega t).$$

Application of the multimodal approach for the forced vibration analysis leads to the following solution:

(26) $$q=a_{C}\cos\left(\omega t\right)+a_{S}\sin\left(\omega t\right),$$

where parameter *ω* is angular frequency, and coefficients **a**_C_ and **a**_S_ are defined as follows:

(27) $$\begin{array}{c} a_{C}={\left\{\left(K-\omega^{2}M\right)+\omega^{2}C\left[{\left(K-\omega^{2}M\right)}^{-1}C\right]\right\}}^{-1}F_{0},\\ a_{S}=\omega {\left(K-\omega^{2}M\right)}^{-1}C{\left\{\left(K-\omega^{2}M\right)+\omega^{2}C\left[{\left(K-\omega^{2}M\right)}^{-1}C\right]\right\}}^{-1}F_{0}. \end{array}$$

In this particular optimization problem, since we consider free vibrations, the damping matrix is equal to zero, and no external forces are involved. Thus, Equation (13) takes the following form:

(28) $$M\ddot{q}(t)+Kq(t)=0.$$

The solution can be expressed as follows:

(29) $$q=q_{a}\sin(\omega t),$$

where **q***_a_* stands for eigenvector. It can be expressed as follows, where *m* is the number of degrees of freedom:

(30) $$q_{a}=\left[q_{a,1},q_{a,2},\ldots ,q_{a,m}\right].$$

Assuming that eigenvalue *λ* is equal to *ω*^2^, Equation (29) can be written as follows:

(31) $$\left(K-\lambda M\right)q_{a}=0.$$

Only the result where **q***_a_* ≠ 0 is considered, so to determine eigenvalues and eigenvectors, the following condition has to be met:

(32) $$\det\left|K-\lambda M\right|=0.$$

There are many approaches to calculating the eigenvalues lambda. For a small number of degrees of freedom, finding roots of the polynomial on the left-hand side of Equation (32) is pretty straightforward. For large systems, more sophisticated methods, such as the QR algorithm [32,33], were developed.

### 2.3. Genetic Algorithm Optimization

Genetic algorithms (GAs) (see [34,35]) are widely used metaheuristic methods for solving complex optimization problems, particularly those involving large search spaces and nonlinear constraints. In one of the authors’ previous studies [36], a GA was applied to optimize the thickness distribution of a plate to maximize the frequency gap between adjacent natural frequencies. For a fixed mode number *k*, the objective function was defined to maximize this gap:

(33) $$\max\Delta \omega_{k}=\max\frac{\omega_{k+1}-\omega_{k}}{\omega_{k+1}},$$

The GA procedure consists of several key steps. First, an initial population of candidate solutions (chromosomes) is generated randomly. Each chromosome encodes a set of genes representing the thickness values of predefined plate regions. The fitness of each individual is evaluated using the objective function, and the population is ranked accordingly. The best-performing individuals undergo genetic operators, including crossover and mutation. Crossover combines genetic material from two parent solutions to produce offspring, while mutation introduces controlled random changes to maintain diversity and prevent premature convergence. After generating offspring, the population is updated by replacing less fit individuals, and the process is repeated until the convergence criteria are met.

**Figure 2:**
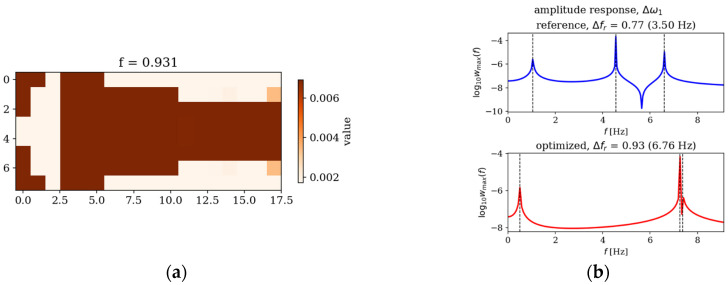
An optimized cantilever plate: (**a**) optimized thickness distribution; (**b**) amplitude–frequency response for a reference (uniform thickness) and optimized plate.

An example result of GA optimization of thickness variation is depicted in Figure 2. A cantilever plate (CFFF) with a 1:2 length-to-width ratio was optimized in terms of the widest relative difference between the first and second natural frequencies. The optimized plate displays a characteristic thickness distribution, which results in widening the frequency gap in comparison with the reference uniform thickness plate.

The population size typically ranges from hundreds of individuals, and multiple generations are often required to achieve satisfactory results. This iterative nature makes GA computationally expensive, as each evaluation involves solving the plate vibration eigenproblem using finite element analysis. Therefore, the integration of machine learning models as surrogate predictors of eigenvalues offers a promising alternative. By replacing repeated FE computations with ANN-based predictions, optimization workflows can be significantly accelerated without compromising accuracy. As in this work, the main focus of optimization is on the side of eigenfrequency values, or more strictly, relative differences between them. Solutions to Equation (32) will be the predicted values.

## 3. Results and Discussion

### 3.1. Introductory Data Analysis

The plate considered in this study is characterized by the following material properties: Young’s modulus *E* = 205 GPa, Poisson’s ratio ν = 0.3, and density ρ = 7850 kg/m^3^. Its dimensions are *L_x_* × *L_y_*, where *L_y_* = 1 m and values of *L_x_* range from 0.5 m to 3.0 m in increments of 0.5 m. The plate thickness ranges between 5 mm and 20 mm. For all dimensions, three types of plate boundary conditions were considered: simply supported on all edges (SSSS), clamped on all edges (CCCC), and cantilever (CFFF). Each plate was discretized into finite elements, with six elements along the *y*-axis (*n_x_* = 6) and a variable number along the *x*-axis (*n_y_* = 6 to 18, depending on the *L_x_* dimension). The plate surface was divided into 36 rectangular subregions, each defined by coordinates (*x_i_*, *y_i_*) and (*x_i_* + *L_x_*/6, *y_i_* + *L_y_*/6) of the bottom-left and upper-right corners, respectively. Each subregion of number *i* was assigned a thickness *h_i_*, where *i* = 1 and *i* = 36 correspond to the bottom-left and top-right corners. Consequently, the input feature set consists of 37 numerical parameters: plate length *L_x_*, 36 thickness values, and an additional categorical feature representing boundary conditions. The categorical feature was converted into a numerical format using one-hot encoding [37].

A dataset of 54,000 samples was generated, corresponding to 3000 samples for each of the 18 combinations of boundary conditions and plate lengths. Thickness distributions were randomly generated using a uniform distribution, followed by two-dimensional cubic spline interpolation to ensure smooth variation between adjacent elements. The output comprises natural frequencies in ascending order; in this study, only the three lowest frequencies (*f*_1_–*f*_3_) are considered.

**Figure 3:**
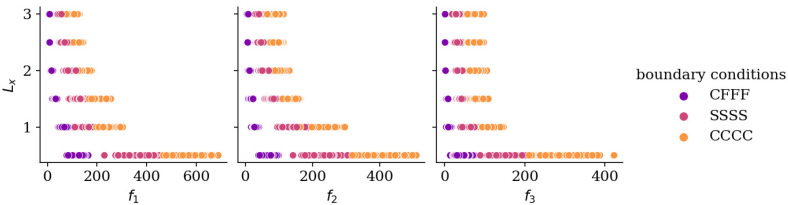
Natural frequencies *f*_1_–*f*_3_ vs. length *L_x_* of the plate for various boundary conditions, training set.

**Figure 4:**
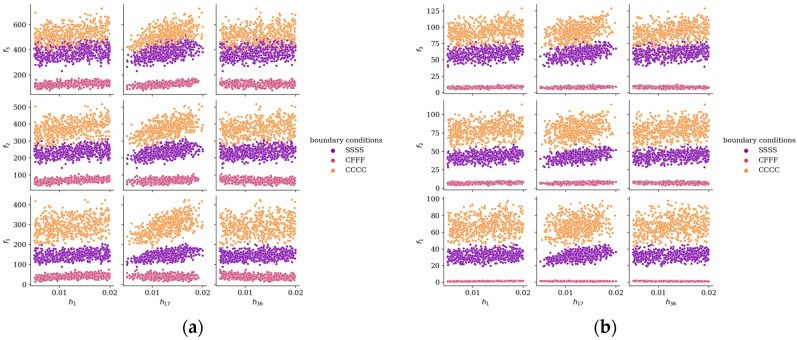
Relationship between natural frequencies *f*_1_–*f*_3_ and thickness of elements 1, 17, and 36 for various boundary conditions: (**a**) *L_x_*/*L_y_* = 1/2; (**b**) *L_x_*/*L_y_* = 3/1.

Figure 3 illustrates the relationship between the three lowest natural frequencies and plate length *L_x_* for different boundary conditions, based on a sample of 5000 configurations. For uniform rectangular plates, natural frequencies are inversely proportional to the square of their length [23]. Figure 4 shows the dependence of frequencies *f*_1_–*f*_3_ on the thickness of selected subregions: *h*_1_, *h*_17_, and *h*_36_ (bottom-left, center-right, and top-right subregions of the plate) for two aspect ratios: *L_x_*/*L_y_* = 1/2 and *L_x_*/*L_y_* = 3/1. For CFFF plates, frequencies exhibit mixed trends, either increasing or slightly decreasing with thickness, whereas for SSSS and CCCC plates, frequencies consistently increase as thickness grows.

### 3.2. ANN Models Development

#### 3.2.1. Development of the Baseline ANN Model

The algorithms presented in this work—finite element modeling as well as ANN training and validation—were implemented in Python 3.11.1. The development of the ANN model was carried out using the scikit-learn machine learning library. As an artificial neural network with hidden layers can be considered a black box with predictive capabilities, there are no strict rules for selecting its topology. Moreover, numerous hyperparameters must be tuned, including both preprocessing and regressor settings.

First, the input features were divided into categorical (boundary conditions) and numerical (plate length and thickness values). Categorical data were converted into a numerical format using one-hot encoding, which creates binary columns for each unique category. Because the influence of plate length and thickness differs, two separate polynomial transformers were applied—of up to order 5 for length and order 2 for thickness. For the regressor, several ANN topologies were tested, ranging from two to four hidden layers with 6 to 96 neurons per layer. Two activation functions were considered: hyperbolic tangent (Tanh) and rectified linear unit (ReLU). Model performance was evaluated using the root mean squared error (RMSE) as the primary metric.

An exhaustive grid search over the specified parameter space revealed the best configuration: two hidden layers with 96 neurons each, polynomial transformers of order 5 and 1 for length and thickness, respectively, and ReLU activation. During parameter tuning, cross-validation was performed with a test set size of 20% for 9000 samples. The results were then analyzed in detail, including the influence of training set size and plate parameters.

**Figure 5:**
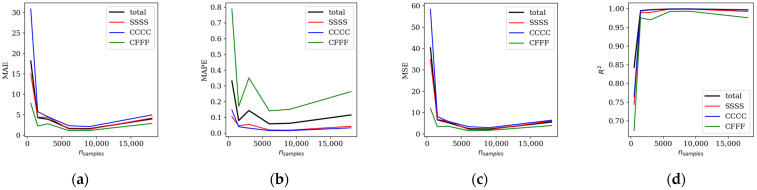
Influence of the dataset size on: (**a**) MAE; (**b**) MAPE; (**c**) MSE; (**d**) *R*^2^ coefficient (abbreviations explanation in the text)—baseline model.

The dataset size was examined by randomly selecting subsets of *n_s_* = 500, 1500, 3000, 6000, 9000, and 18,000 samples from the full dataset. The influence of training set size on model performance was assessed using mean absolute error (MAE), mean absolute percentage error (MAPE), mean squared error (MSE), and the coefficient of determination (*R*^2^). These results are summarized in Table 1 and illustrated in Figure 5 for all boundary conditions, as well as for each case individually (SSSS, CCCC, and CFFF).

**Figure 6:**
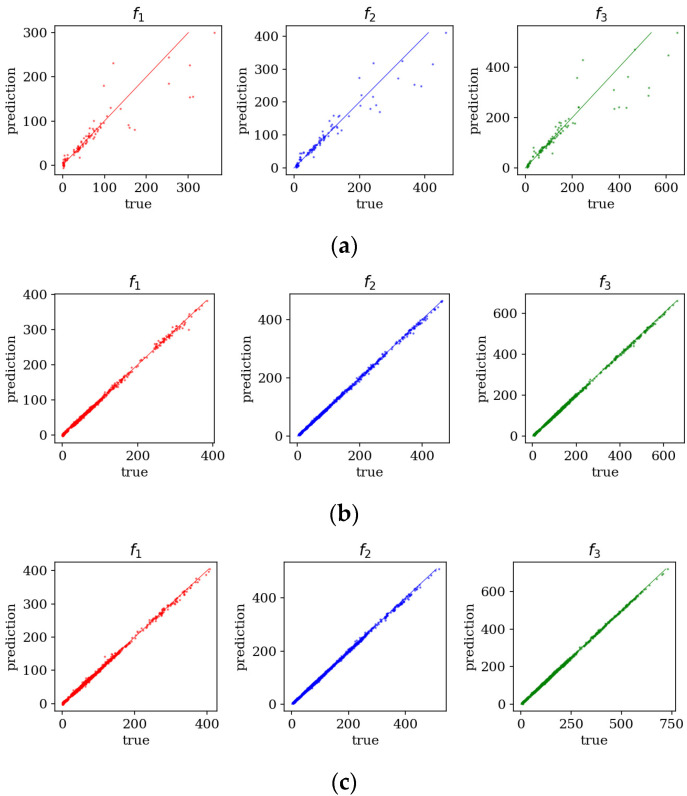
Predicted vs. true values of frequencies *f*_1_–*f*_3_, for various sample amounts: (**a**) 500 samples; (**b**) 6000 samples; (**c**) 9000 samples.

Figure 6 compares predicted and true values of the three lowest natural frequencies for different dataset sizes. Across all boundary conditions, models trained on 6000 and 9000 samples achieved the highest accuracy, with only minor differences between error metrics. Consequently, further analysis focuses on the model trained with 9000 samples.

An important question is whether prediction accuracy varies among the three lowest frequencies and across different plate lengths. Table 2 presents MAPE and *R*^2^ values for each frequency separately under all boundary conditions. The results indicate that accuracy is lowest for the first fundamental frequency, while differences between the second and third frequencies are relatively small.

To explore this further, Table 3 shows how MAPE depends on both boundary conditions (categorical) and plate length (numerical). For SSSS and CCCC plates, the increase in MAPE with plate length is negligible. However, for CFFF plates, the error grows significantly as plate length increases. These findings confirm that prediction efficiency deteriorates for cantilever plates, particularly for elongated geometries.

#### 3.2.2. Boundary–Condition-Specific Model Optimization

The initial ANN model demonstrated strong performance for plates with SSSS and CCCC boundary conditions but showed noticeable accuracy degradation for CFFF plates, particularly for elongated geometries. To address this limitation, an improved modeling strategy was developed by training separate networks for each boundary condition rather than using a single unified model. This approach aims to reduce the complexity introduced by categorical features and allow the network to specialize in capturing the unique vibrational characteristics associated with each support type.

**Figure 7:**
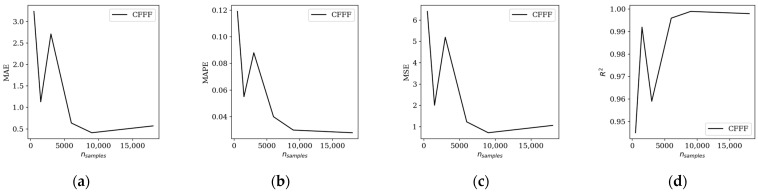
Influence of the dataset size on: (**a**) MAE; (**b**) MAPE; (**c**) MSE; (**d**) *R*^2^ coefficient (abbreviations explanation in the text)—boundary specific model.

For the cantilever case (CFFF), which previously exhibited the highest prediction errors, the improved model was trained on subsets of varying sizes: 500, 1500, 3000, 6000, 9000, and 18,000 samples. Performance was evaluated using MAE, MAPE, MSE, and *R*^2^ metrics, as summarized in Table 4 and illustrated in Figure 7. Compared to the baseline model, the specialized network achieved a substantial reduction in error, particularly for larger training sets. For example, with 9000 samples, MAPE decreased from 0.1416 in the baseline model to 0.030, and *R*^2^ improved from 0.993 to 0.999, indicating a very good correlation between predicted and true values.

Further analysis examined prediction accuracy for individual frequencies and plate lengths. Table 5 shows that the improved model significantly reduced discrepancies for the first fundamental frequency, which had previously been the most challenging to predict. Similarly, Table 6 demonstrates that while error still increases with plate length, the growth rate is much lower than in the baseline model, confirming enhanced robustness for elongated plates.

**Figure 8:**
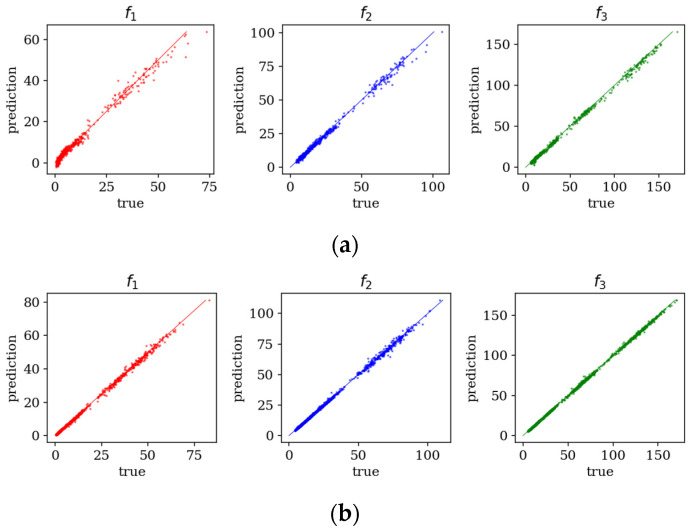
Predicted vs. true values of frequencies *f*_1_–*f*_3_ of CFFF plate for 9000 samples: (**a**) basic model; (**b**) improved model.

Figure 8 provides a visual comparison of predicted versus true frequencies for the baseline and improved models. The improved approach exhibits tighter clustering around the diagonal, reflecting higher prediction fidelity. These results validate the hypothesis that boundary–condition-specific training improves generalization and accuracy, particularly for complex support configurations such as cantilever plates.

In summary, this refinement highlights the importance of model specialization in structural dynamics applications. By reducing feature heterogeneity and focusing on condition-specific patterns, ANN models can achieve superior performance without requiring excessively large datasets. This strategy can be extended to other structural elements and support conditions.

### 3.3. Discussion

The results confirm that ANNs can serve as efficient surrogate models for predicting vibration frequencies of thin plates with variable thickness. Compared to traditional FE analysis, the proposed approach significantly reduces computational effort while maintaining high accuracy. However, prediction accuracy depends on boundary conditions and plate geometry. While the baseline ANN achieved excellent accuracy for SSSS and CCCC plates, its performance deteriorated for CFFF plates, particularly for elongated geometries. Training separate models for each boundary condition significantly improved accuracy, reducing MAPE for cantilever plates from 0.1416 to 0.030 and increasing *R*^2^ to nearly 0.999. These improvements demonstrate that boundary–condition-specific training is a practical strategy for enhancing robustness.

Overall, these results highlight the need for tailored machine learning strategies in structural dynamics applications, especially when dealing with complex support conditions and non-uniform geometries.

## 4. Conclusions

This study demonstrates that ANNs can effectively predict vibration frequencies of thin plates with variable thickness, offering a computationally efficient alternative to FE analysis in optimization workflows. From a computational standpoint, generating the full FEM dataset of 54,000 cases required approximately 35 min on the hardware used in this study, whereas hyperparameter tuning and training on 9000 samples took about 540 s. Once trained, the ANN can predict frequencies for thousands of plate configurations within a few seconds, i.e., orders of magnitude faster than repeatedly solving the eigenvalue problem with FEM for the same number of cases. Considering that a single genetic-algorithm-based optimization may involve several thousand eigenvalue evaluations for one plate length and one boundary condition, replacing FEM calls with ANN predictions can substantially accelerate such optimization workflows without sacrificing accuracy.

The proposed approach, therefore, significantly reduces processing time while maintaining high accuracy, particularly when models are tailored to specific boundary conditions. These findings suggest that ML-based surrogate models can accelerate structural design processes and enable more advanced optimization strategies for lightweight, vibration-resistant structures in mechanics and civil engineering. Future research will focus on extending the approach to other plate types and support conditions and on exploring more advanced architectures, such as convolutional neural networks (CNNs).

## Figures and Tables

**Table 1:** MAE, MAPE, MSE, and *R*^2^ vs. size of the set for all boundary conditions.

*n_s_*	Overall				SSSS				CCCC				CFFF			
	MAE	MAPE	MSE	*R* ^2^	MAE	MAPE	MSE	*R* ^2^	MAE	MAPE	MSE	*R* ^2^	MAE	MAPE	MSE	*R* ^2^
500	18.171	0.331	40.334	0.843	15.008	0.105	34.914	0.744	30.88	0.147	58.299	0.764	7.806	0.789	12.000	0.674
1500	4.283	0.080	6.631	0.995	4.401	0.047	6.815	0.989	5.772	0.041	8.198	0.994	2.268	0.169	3.425	0.976
3000	3.904	0.143	5.383	0.997	4.402	0.056	6.088	0.990	4.492	0.031	5.995	0.997	2.780	0.351	3.679	0.970
6000	1.642	0.059	2.509	0.999	1.518	0.020	2.173	0.999	2.317	0.016	3.448	0.999	1.119	0.142	1.591	0.993
9000	1.592	0.062	2.327	0.999	1.466	0.019	2.055	0.999	2.114	0.016	2.978	0.999	1.163	0.151	1.703	0.993
18,000	3.988	0.115	5.592	0.997	4.196	0.043	6.076	0.993	4.956	0.033	6.464	0.997	2.874	0.264	3.973	0.976

**Table 2:** MAPE and *R*^2^ score for frequencies *f*_1_–*f*_3_ for all considered boundary conditions separately.

f	Overall		SSSS		CCCC		CFFF	
	MAPE	*R* ^2^	MAPE	*R* ^2^	MAPE	*R* ^2^	MAPE	*R* ^2^
*f*_1_	0.1286	0.9989	0.0274	0.9976	0.0200	0.9987	0.3346	0.9870
*f*_2_	0.0298	0.9994	0.0147	0.9994	0.0143	0.9991	0.0598	0.9951
*f*_3_	0.0285	0.9996	0.0139	0.9996	0.0127	0.9996	0.0584	0.9978

**Table 3:** MAPE score for all considered boundary conditions and length *L_x_* values, bc—for specified boundary conditions, bc + len—for specified boundary conditions and length *L_x_*.

Boundary Conditions	*L_x_*	Overall	bc	bc + len
SSSS	0.5	0.0589	0.0199	0.0131
	1.0			0.0170
	1.5			0.0180
	2.0			0.0176
	2.5			0.0225
	3.0			0.0305
CCCC	0.5	0.0589	0.0160	0.0120
	1.0			0.0174
	1.5			0.0170
	2.0			0.0170
	2.5			0.0153
	3.0			0.0192
CFFF	0.5	0.0589	0.1416	0.0404
	1.0			0.0572
	1.5			0.1026
	2.0			0.1395
	2.5			0.2081
	3.0			0.2995

**Table 4:** MAE, MAPE, MSE, and *R*^2^ vs. the size of the set.

*n_s_*	CFFF			
	MAE	MAPE	MSE	*R* ^2^
500	3.236	0.119	6.411	0.945
1500	1.131	0.055	2.005	0.992
3000	2.711	0.088	5.198	0.959
6000	0.637	0.040	1.224	0.996
9000	0.411	0.030	0.714	0.999
18,000	0.570	0.028	1.058	0.998

**Table 5:** MAPE and *R*^2^ score for frequencies *f*_1_–*f*_3_ for CFFF boundary conditions.

f	CFFF	
	MAPE	*R* ^2^
*f*_1_	0.0517	0.9986
*f*_2_	0.0208	0.9988
*f*_3_	0.0185	0.9997

**Table 6:** MAPE score for CFFF boundary conditions versus *L_x_* values.

*L_x_*	MAPE
0.5	0.0165
1.0	0.0211
1.5	0.0227
2.0	0.0283
2.5	0.0371
3.0	0.0553

## Data Availability

The original contributions presented in this study are included in the article. Further inquiries can be directed to the corresponding author.
